# Pediatric admissions that include intensive care: a population-based study

**DOI:** 10.1186/s12913-018-3041-x

**Published:** 2018-04-10

**Authors:** Ibinabo Ibiebele, Charles S. Algert, Jennifer R. Bowen, Christine L. Roberts

**Affiliations:** 1 Clinical and Population Perinatal Health Research, Kolling Institute, Northern Sydney Local Health District, Sydney, Australia; 2 Sydney Medical School Northern, University of Sydney, Sydney, Australia; 3 Department of Neonatology and Paediatrics, Royal North Shore Hospital, Sydney, Australia

**Keywords:** Intensive care, Critical care, Pediatrics, Children, Hospitalization, Mortality

## Abstract

**Background:**

Pediatric admissions to intensive care outside children’s hospitals are generally excluded from registry-based studies. This study compares pediatric admission to specialist pediatric intensive care units (PICU) with pediatric admissions to intensive care units (ICU) in general hospitals in an Australian population.

**Methods:**

We undertook a population-based record linkage cohort study utilizing longitudinally-linked hospital and death data for pediatric hospitalization from New South Wales, Australia, 2010–2013. The study population included all new pediatric, post-neonatal hospital admissions that included time in ICU (excluding neonatal ICU).

**Results:**

Of 498,466 pediatric hospitalizations, 7525 (1.5%) included time in an intensive care unit – 93.7% to PICU and 6.3% to ICU in a general (non-PICU) hospital. Non-PICU admissions were of older children, in rural areas, with shorter stays in ICU, more likely admitted for acute conditions such as asthma, injury or diabetes, and less likely to have chronic conditions, receive continuous ventilatory support, blood transfusion, parenteral nutrition or die.

**Conclusions:**

A substantial proportion of children are admitted to ICUs in general hospitals. A comprehensive overview of pediatric ICU admissions includes these admissions and the context of the total hospitalization.

## Background

Fortunately, relatively few children require intensive care during a hospital admission but those who do are likely to suffer high morbidity and mortality rates and represent a disproportionate burden on pediatric services [1]. Children admitted to intensive care units (ICUs) have a heterogenous mix of conditions including admissions for acute illness such as asthma or due to external causes such as injury as well as relatively rare problems such as metabolic diseases and complex congenital malformations [2]. Ideally, children requiring intensive care are cared for in specialist pediatric ICUs (PICU) in children’s hospitals, but this is not always feasible. Access to PICUs is particularly relevant in countries such as Australia, the United States and Canada with large land masses and highly variable population densities.

Pediatric ICUs may care for both neonates (≤28 days of age) and older children, with 9% of PICU admissions in Australia in 2012 being neonates [2]. However, in contrast to the large body of literature on neonatal admissions and outcomes, [3, 4] the post-neonatal and childhood use of ICUs has been infrequently reported and is usually based on specialist PICU registry data that do not place the ICU admissions within the broader context of hospital admissions [5–8]. Admissions to an ICU outside of a children’s hospital are generally not included in studies drawn from PICU registry data and PICU registry studies have rarely included outcome data beyond discharge from the PICU or hospital [9].

A comprehensive description of new pediatric admissions which result in ICU stays should include general hospital ICUs as these may be the first step in treatment, or the only treatment site in cases which resolve without recourse to a children’s hospital. Other than a needs assessment for south-east England in 1997, [10] the frequency and outcomes of population-level pediatric admissions to ICUs in general hospitals has not been reported. Therefore, the aim of this study was to use linked population data to describe pediatric hospital admissions which result in time in ICU by type of ICU (specialised PICU at children’s hospitals and ICU admission at other hospitals), in an Australian population.

## Methods

We undertook a population-based cohort study in New South Wales (NSW) Australia using public hospital data from 2010 to 2013. NSW is the most populous Australian state with ~ 7.7 M residents, 32% of the national population. The state has an area of over 800,000 km^2^, with population densities ranging from > 4000 people per km^2^ in coastal metropolitan cities to < 0.1/1000 km^2^ in inland/remote communities [11]. Pediatric care is regionalised, [12, 13] with more than 150 public hospitals providing acute care ranging from small rural hospitals with care provided by general practitioners to tertiary general and 3 specialist children’s hospitals. There is a specialised retrieval service for critically ill children [14]. Private hospitals provide a very limited range of acute pediatric and emergency care.

Data were obtained from the NSW Admitted Patient Data Collection, a census of all public and private inpatient admissions to NSW hospitals. Diagnoses and procedures are coded according to the 10th revision of the International Classification of Disease, Australian Modification (ICD-10-AM) and the Australian Classifications of Health Interventions (ACHI). The admission records were longitudinally linked, enabling identification of new post-neonatal admissions for each child [15]. Deaths outside of hospital were identified using linked data from the NSW Registry of Births, Deaths and Marriages. The study was approved by the NSW Population and Health Services Research Ethics Committee (2002/12/430).

The study population included all new pediatric, post-neonatal hospital admissions (> 28 days and up to the 16th birthday) that included time in ICU (> 0 ICU hours). No restrictions were placed on time in ICU as all admissions including those awaiting transfer were of interest. Although neonates contribute to PICU admissions, [2] only children re-admitted in the post-neonatal period were included as neonatal and pediatric ICU hours cannot be differentiated in the data and our interest was PICU, rather than NICU. Children could have multiple new hospital admissions with ICU hours during the study period; each hospital admission was included in the study. For example, if a child was admitted to ICU at a non-children’s hospital and subsequently transferred to a children’s hospital PICU, this was counted as two separate admissions. Readmissions to ICU during a single hospital stay could not be identified separately as only total ICU hours for each hospital admission is recorded.

The primary aim was to report the number of pediatric hospital admissions that included PICU admissions in a specialised Children’s Hospital or ICU admissions at other public general hospitals (classified as tertiary, other urban, and rural hospitals) and to compare the characteristics and outcomes for children to PICUs and general ICUs. We compared admissions to specialised PICUs and general ICUs on: age at hospital admission (≥29 days to < 1 year, ≥1 to < 5 years, ≥5 to < 10 years, ≥10 to < 16 years), gender, urban/rural residence; source of hospital admission: (emergency, transfer from other hospital, physician referral, hospital outpatient clinic, other/unknown); total ICU hours (median and interquartile range [IQR], hour categories); total length of hospital stay in days until discharge or transfer (median, IQR); admission outcome (death, discharge, transfer). Hospital mortality is reported as deaths per 100 pediatric admissions that included ICU. The use of support procedures including parenteral nutrition, blood transfusion or continuous ventilatory support was tabulated. Procedure codes used to identify continuous ventilatory support are provided in Appendix.

We categorized the principal diagnosis code recorded for each hospital admission according to the ICD10-AM chapters as: diseases of the respiratory system (including acute upper and lower respiratory tract infections and asthma), congenital malformations, diseases of the nervous system (including epilepsy), endocrine and metabolic diseases (including diabetes), diseases of the circulatory system, neoplasms, infectious and parasitic diseases, diseases of the digestive system (including appendicitis), musculoskeletal disorders, complications of medical care, injury and external causes of morbidity and mortality. We also examined the first five diagnosis fields to identify admissions of children with chronic conditions according to the Hardelid classification categories [16] (eg neurosensory impairment, congenital abnormalities etc).

Mortality rates at one year post-discharge were calculated per child who had an ICU admission resulting in discharge home or transfer during 2010–2012. Additional mortality data were not available in the current linkage for the 2013 admissions. If a child had more than one admission with ICU hours, the mortality rate was calculated based on death within one year after the last discharge.

### Analysis

The admission data were whole population data and not “sampled” data so no sampling error testing was performed. Any differences in proportions represent real population differences for the study period. Trend in use of services was assessed using the Cochran-Armitage test.

## Results

From 2010 through 2013 there were 498,466 hospital admissions of children aged ≥29 days to < 16 years in NSW. Among these admissions there were 7525 post-neonatal pediatric public hospital admissions that included time in an ICU, or an average of 1881 per annum. Thus the rate of any ICU time per hospital admission was 15.1 per 1000. These admissions were of 6124 children: 5245 (85.6%) only had one such admission, 614 (10.0%) had two and 265 (4.3%) had three or more. Of all the admissions, 7053 (93.7%) were in hospitals with a specialist pediatric intensive care unit (PICU) and 472 (6.3%) admissions were to an ICU in a general public hospital (non-PICU). The PICU admissions were generally younger children and transfers from another facility (Table 1). Pediatric admissions with ICU time in general hospitals were more common in rural areas, over three-quarters were emergency hospitalizations and 53% were 10 years or older. Of the hospital admissions with hours in a PICU, 27.8% were categorized as surgical admissions compared to 24.6% at general hospitals. Both length of stay in ICU and total length of hospital stay were longer for admissions to a specialist PICU, and had a higher hospital mortality rate than admissions in general ICUs (2.9% vs 1.3%). Time spent in ICU differed markedly; most commonly (70.6%) ≤24 h in general hospitals and commonly (26.2%) > 96 h in PICUs. For children transferred from general hospitals, the destination hospital was unrecorded for 19 of 134 (14.2%) transfers. Among the 115 where the destination was recorded, 96 (83.5%) were transfers to a children’s hospital.

**Table 1:** Characteristics of pediatric (post-neonatal) hospital admissions that included time in an ICU^a^ 2010–2013

Hospital admission episode characteristic	PICU hospitals *N* = 7053 n (%)	Non- PICU hospitals *N* = 472 n (%)	*P*-value ^b^
Age			
1 to < 12 months	2292 (32.5)	42 (8.9)	< 0.001
1–4 years	2348 (33.3)	94 (19.9)	< 0.001
5–9 years	1207 (17.1)	85 (18.0)	0.62
10–15 years	1205 (17.1)	251 (53.2)	< 0.001
Sex			
Male	3928 (55.7)	245 (51.9)	0.11
Female	3125 (44.3)	227 (48.1)	0.11
Residence			
Urban	4973 (70.7)	74 (15.7)	< 0.001
Rural	2063 (29.3)	397 (84.3)	< 0.001
Hospital type			
Tertiary	7053 (100)	57 (12.1)	< 0.001
Other urban	0 (0)	34 (7.2))	< 0.001
Regional	0 (0)	381 (80.7)	< 0.001
Source of hospital admission			
emergency admission	1711 (24.3)	363 (76.9)	< 0.001
transfer admission	2289 (32.4)	52 (11.0)	< 0.001
physician referral	1346 (19.1)	41 (8.7)	< 0.001
outpatient clinic	1408 (20.0)	7 (1.5)	< 0.001
other/unknown	297 (4.2)	9 (1.9)	0.01
ICU hours (median, IQR)	47 (24–100)	15 (6–27)	< 0.001^c^
ICU hours			
≤ 24 h	1913 (27.1)	333 (70.6)	< 0.001
> 24 to ≤48 h	1745 (24.7)	88 (18.6)	0.003
> 48 to ≤96 h	1550 (22.0)	30 (6.4)	< 0.001
> 96 h	1845 (26.2)	21 (4.5)	< 0.001
Days in hospital (median, IQR)	6 (4–12)	2 (1–5)	< 0.001^c^
Admission outcome			
Death	203 (2.9)	6 (1.3)	0.04
Discharge	6349 (90.0)	332 (70.3)	< 0.001
Transfer	497 (7.1)	134 (28.4)	< 0.001
to children’s hospital	46 (9.3)	96 (71.6)	< 0.001
to other hospital	390 (78.5)	19 (14.2)	< 0.001
not recorded	61 (12.3)	19 (14.2)	< 0.56

^a^per admission (including time in ICU) until discharge home or transfer to another hospital

^b^Test of two proportions (row values)

^c^Wilcoxon test

Table 2 shows the distribution of primary diagnoses associated with hospital admissions that included ICU and the frequency of any chronic conditions, by PICU/non-PICU hospital status. Respiratory illnesses (predominantly acute respiratory infections) and congenital anomalies were the most common reasons for admission in PICU hospitals. Injury and external causes, respiratory illnesses (especially asthma) and endocrine/metabolic disorders (primarily diabetes) were the most common admitting diagnoses in non-PICU hospitals. Epilepsy was the most common neurological disorder (3.4% of PICU admissions, 4.7% of non-PICU), malignant brain tumours were the most common neoplasms (2.2% PICU, 0.2% non-PICU) and diabetes was the most common endocrine disorder (1.5% PICU, 15.0% non-PICU). Diabetes was the second most common admitting diagnosis after injury at non-PICU hospitals and affected females disproportionately: 18% versus 12% of admissions for males. Injury-related causes accounted for 8.6% of admissions overall, but was proportionally higher (29.0%) in general ICU admissions than for PICU admissions (7.1%).

**Table 2:** Principal diagnosis for NSW pediatric post-neonatal hospital admissions which included time in an ICU 2010–2013

Reason for admission to hospital	PICU hospitals N = 7053 n (%)	Non-PICU hospitals N = 472 n (%)	*p*-value ^c^
Principal diagnosis			
Respiratory illnesses	2012 (28.5)	121 (25.6)	0.18
Acute infections	1341 (19.0)	48 (10.2)	< 0.001
Asthma	348 (4.9)	56 (11.9)	< 0.001
Congenital anomalies	1599 (22.7)	10 (2.1)	< 0.001
Neurological disorders	597 (8.5)	30 (6.4)	0.11
Injury and external cause	501 (7.1)	137 (29.0)	< 0.001
Neoplasms	534 (7.6)	5 (1.1)	< 0.001
Endocrine/metabolic diseases	197 (2.8)	77 (16.3)	< 0.001
Circulatory system disorders	267 (3.8)	18 (3.8)	0.98
Infections (except respiratory)	255 (3.6)	11 (2.3)	0.14
Musculoskeletal disorders	243 (3.5)	5 (1.7)	0.005
Appendicitis	41 (0.6)	6 (1.3)	0.07
Complications of medical care	152 (2.2)	^a^ (0.9)	0.05
Other miscellaneous	655 (9.3)	48 (10.2)	0.52
Chronic disease conditions ^b^			
none	1561 (22.1)	158 (33.5)	< 0.001
1	3638 (51.6)	271 (57.4)	0.01
≥ 2	1854 (26.3)	43 (9.1)	< 0.001

^a^Cell size < 5

^b^Categorized according to Hardelid

^c^Test of two proportions (row values)

Chronic disease conditions were common in children admitted to an ICU. At least one chronic condition was documented in 77.2% of admissions; 77.9% of PICU and 66.5% of the general ICU. Among the 5806 admissions with a chronic condition, 1897 (32.7%) had a second chronic condition. The chronic conditions were frequently (36.7%) concurrent with a congenital anomaly.

Table 3 shows the five most common primary admission diagnoses by age group and by PICU/non-PICU hospital status. Respiratory disease was the most common reason for all age and hospital groups except among older children at general hospitals. A congenital anomaly, usually a cardiovascular malformation, was the second most common reason for hospital admission among children admitted to a PICU, with 31% of PICU admissions < 1 year old identified as having a cardiovascular anomaly. Past one year of age, neurological conditions increased in frequency. The majority of admissions at non-specialist ICUs were of children aged ≥5 years and the most common reason in this age group was an injury/external cause with poisoning/toxic exposure being the most common injury within this subgroup.

**Table 3:** NSW hospital admissions that include ICU 2010–2013: principal admission diagnosis by child age and hospital type

	PICU hospitals		Non-PICU hospitals	
Age	Reason for admission	n (%)	Reason for admission	n (%)
> 28 days to < 12 months	1. respiratory	869 (37.9)	1. respiratory	20 (47.6)
	2. congenital anomaly	760 (33.1)	2. other (perinatal-related)	7 (16.7)
	3. other infectious	115 (5.0)	3 injury	5 (11.9)
	4. neoplasm	76 (3.3)	4. congenital anomaly	^a^ (4.8)
	5. neurological	69 (3.0)	5. other infectious	^a^ (4.8)
≥1 year to < 5 years	1. respiratory	672 (28.6)	1.respiratory	38 (40.4)
	2. congenital anomaly	509 (21.7)	2. injury	18 (19.2)
	3. neurological	253 (10.8)	3 neurological	14 (14.9)
	4. injury	211 (9.0)	4. endocrine disorders	9 (9.6)
	5. neoplasm	207 (8.8)	5. circulatory	^a^ (3.2)
≥5 years to < 16 years	1.respiratory	471 (19.5)	1. injury	114 (33.9)
	2. congenital anomaly	330 (13.7)	2. endocrine disorders	68 (20.2)
	3. neurological	275 (11.4)	3 respiratory	63 (18.8)
	4. neoplasm	251 (10.4)	4. circulatory	15 (4.5)
	5. injury	235 (9.7)	5. neurological	15 (4.5)

^a^cell size < 5 admissions

The mortality rate during an admission increased with increasing age at admission. For infants ≥29 days to < 1 year the rate was 2.2%, for children ≥1 year to < 5 years it was 2.7% and for children ≥5 to < 16 years it was 3.1%. This was partly due to the increase in admissions for external causes for older children, as external cause admissions had an overall mortality rate of 4.2%. Admissions for acute respiratory infection had a mortality rate of only 0.7%. Admissions which included a chronic condition had a mortality rate of 2.8%.

Overall 2.4% of children in PICU received parenteral nutrition; parenteral nutrition was rarely used for children in general hospital ICUs. Use of parenteral nutrition varied greatly by length of stay in PICU. For children in a PICU with stays < 48 h, only 0.9% were started on parenteral nutrition. This rose to 2.2% for stays of 2–5 days and to 8.0% for stays ≥6 days. Continuous ventilatory support was utilized for 42.2% of children at specialist PICU and for 17.6% of other hospitals. Continuous ventilatory support was provided for 53.5% of all children aged < 1 year but this declined to 30.2% among children aged 10–15 years. A blood transfusion was received by 20.9% of children at PICU hospitals and 5.6% of children in general hospitals. The transfusion rate was highest (25.4%) for infants < 1 year, dropping to 19.1% at 1–4 years, 15.3% at 5–9 years and 15.5% at 10–15 years. The per annum rate of transfusion dropped each year of the study period. The overall rate was 23.2% in 2010 but had dropped to 17.8% in 2013 (trend *P* < 0.001).

Table 4 shows the one year post-discharge mortality rates (based on last discharge) by primary diagnosis and age at admission for children with hours in ICU during 2010–2012. There were 4439 children discharged alive from ICU. The overall post-discharge mortality among children who were discharged alive was 98 (2.2%); 2.3% after PICU and 1.5% after non-PICU admissions. The PICU rate was particularly skewed by the group of children aged ≥1 year with a neoplasm, who had a 1 year mortality rate of 11.7%. Excluding the children with neoplasms, the one year mortality rate after discharge from a PICU was 1.7%.

**Table 4:** Mortality rate, by age at admission, at one year post-discharge^a^ for children with a hospital admission that included ICU, 2010–2012

	Mortality per 100 infants	
Primary admitting diagnosis	Age ≥ 29 days to < 1 year	Age ≥ 1 year to < 16 years
Acute respiratory infection	1.2	1.1
Other non-asthma respiratory ^b^	3.4	5.2
Congenital anomaly	0.8	0.9
Neurological disorder	5.0	1.8
Injury	0.0	0.8
Neoplasm	0.0	11.6
Endocrine/metabolic	12.5	2.6
Other diagnosis	3.0	1.9

^a^if more than one ICU admission for a child, then after the last such admission

^b^mainly aspiration pneumonia

## Discussion

The large majority of new post-neonatal pediatric ICU admissions were in tertiary children’s hospitals (93.7%) but a substantial group of children (6.3%, or more than 110 admissions per year) were admitted to hospitals with general ICUs. This is important as it highlights the need for both medical and nursing staff in general ICUs, particularly in regional and non-tertiary hospitals, to maintain high level skills in the care of pediatric admissions. Although the case mix is somewhat different in general hospitals, with children more likely to be older and presenting with an injury, diabetes or asthma, skills required to manage these patients and their families differ from those for adults. For example, medical care may differ in equipment required, approach to fluid management and medication doses suitable for the child’s weight and general care of the child requires an understanding of the developmental stage and abilities of the child, the importance of including the family in care and decision making and child protection issues.

The strengths of this study include the use of whole-population data that provides a comprehensive overview of all new post-neonatal childhood hospitalizations that include ICU admission. Longitudinal record linkage of multiple sources allows assessment of outcomes including multiple admissions, transfers and deaths. The record linkage is undertaken independent of the research using best practice privacy-preserving principles and achieves high (> 95%) linkage rates [15, 17]. Validation studies comparing routinely collected hospital data with medical records show excellent levels of agreement with the medical record and low rates of missing data [18–21]. There is high specificity (> 99%) in the identification of diagnoses and procedures, indicating few false positive reports, although under-reporting for some conditions does occur. However, with the exception of newborns, most validation studies have been in general hospital populations and so primarily among adult admissions [18–21]. Studies based on PICU registries have detailed clinical data for the ICU admissions but not for the entire hospital stay [5–8]. Consequently registry studies may not capture the full burden on health services, as children may have prolonged stays in hospital after being transferred out of an ICU. Compared to PICU registries, our hospital data lack clinical detail and the temporal sequence of events during hospitalization, but it is well suited to a descriptive study of hospital admissions [22]. However, the burden of chronic disease and healthcare utilization described in this study may be an underestimate as new post-neonatal pediatric hospitalizations excluded admissions extending from the neonatal to pediatric period as well as admissions that commenced prior to the start of the study period.

The Report of the Australian and New Zealand Paediatric Intensive Care Registry for 2012 reported 2500 pediatric ICU admissions in 2012 [2]. This is higher than the average of 1881 new post-neonatal admissions in our study. However, the Registry data include neonates admitted to a PICU and separately counts multiple admissions to PICU during a single hospital episode but does not include all general ICUs in NSW. The results reported in the current study are per hospital admission rather than per ICU admission. Like Maybloom et al., [10] our data only counted new post-neonatal admissions. Infants who stayed in hospital for months following their birth could not be counted as new admissions to ICU until after a discharge home. The different reporting basis is also reflected in the reasons for admission, which are for the hospital admission rather than the admission to ICU. Post-operative complications, for instance, are rarely the reason for an initial hospital admission. A study which examined length of stay in Australian and New Zealand PICUs reported that the modal stay was 18–24 h which accords with the PICU results of this study [23].

Estimates of the burden of chronic disease can vary, depending on how chronic conditions are identified (including the number of available diagnosis fields) and coded [16, 24]. Similar to our rate of chronic conditions, a study utilizing the US Virtual Pediatric Intensive Care Unit Systems (VPS) database for 2008 reported 72% of pediatric patients had a chronic condition [5]. However, Typpo et al. reported the frequency of chronic conditions was 52%, based on secondary diagnoses in the VPS 2004–5, with cardiac conditions most common [8].

PICU admissions for diabetes have been reported to be rising in the UK and the incidence of diabetes admissions at non-PICU hospitals in our study underlines the frequency of presentation of children with Type 1 diabetes requiring high level medical care outside of areas with specialist PICU care [25]. Like the present study, Burns et al report more females than males (56% versus 44%) among acute admissions for diabetes, noting their increased risk for developing diabetic ketoacidosis [25]. Factors considered to negatively impact glycaemic control include eating disorders and omission of insulin to affect weight control [25].

Most studies examine mortality in intensive care or hospital. When evaluating follow-up mortality after pediatric admissions, children with neoplasms should be reported separately. Children with neoplasms are much more likely to die outside of an ICU so need to be linked to hospital discharge records and death registration data [26]. The relatively high mortality rate in this condition group can give a misleading impression about overall long term survival for children admitted to ICU.

The data used in this study were collected prior to the publication of a trial of early versus late parenteral nutrition which found that delayed parenteral nutrition resulted in better outcomes [27]. Early parenteral nutrition was not common in this study and this suggests that pediatric ICU practice in NSW had already moved in that direction. The overall transfusion rate of 20.9% was higher than the rate of 14% reported by a 2000 study at a large PICU [28]. In NSW, the rate did drop significantly over the study period, in apparent accord with the results of the Transfusion Strategies for Patients in Pediatric ICU trial which showed that a restrictive transfusion policy is safe [29].

Linked population data can provide a comprehensive picture of all types of pediatric admissions to ICU. Recent studies linking PICU registry data to laboratory and hospital data demonstrate the potential to maximise routinely collected data for answering clinical and health services research questions [30, 31]. The increasing availability of record linkage is facilitating assessment of other long term childhood outcomes including morbidity, mortality, development and educational achievement [32, 33].

## Conclusions

Finally, while most pediatric intensive care occurs in specialist PICU, 6.3% of pediatric intensive care admissions occurred outside of specialist PICUs. General hospital pediatric ICU stays are more likely to involve care of older children with conditions such as asthma and diabetes as well as injuries. Such conditions may warrant priority when planning for maintaining pediatric management skills in non-PICU hospitals.

## Appendix

**Table 5:** Procedure codes used to identify continuous ventilatory support

ACHI Procedure Code	Procedure description
13882–00	Management of continuous ventilatory support, ≤ 24 h
13882–01	Management of continuous ventilatory support, > 24 and < 96 h
13882–02	Management of continuous ventilatory support, ≥ 96 h
13879–00	Continuous ventilatory support, initiation in intensive care unit
22007–00	Endotracheal intubation, single lumen
22007–01	Management of endotracheal intubation, single lumen
92209–01	Management of noninvasive ventilatory support, > 24 and < 96 h
92209–02	Management of noninvasive ventilatory support, ≥ 96 h
92041–00	Continuous negative pressure ventilation
90225–00	Extracorporeal membrane oxygenation
38627–02	Adjustment of cannula for extracorporeal membrane oxygenation

## Abbreviations

[P]ICU: [Pediatric] intensive care unit
ICD-10-AM: International Classification of Disease, Australian Modification
IQR: Interquartile range
NSW: New South Wales
UK: United Kingdom
US: United States
VPS: Virtual Pediatric intensive Care Unit Systems

## References

[1] Namachivayam P, Shann F, Shekerdemian L, Taylor A, van Sloten I, Delzoppo C (2010). Three decades of pediatric intensive care: who was admitted, what happened in intensive care, and what happened afterward. Pediatr Crit Care Med.

[2] Alexander J, Slater A, Woosley J. Report of the Australian and New Zealand Paediatric Intensive Care Registry 2012. Brisbane: Australian and New Zealand Intensive Care Society; 2014.

[3] Chow S, Chow R, Popovic M, Lam M, Popovic M, Merrick J (2015). A selected review of the mortality rates of neonatal intensive care units. Front Public Health.

[4] Lasswell SM, Barfield WD, Rochat RW, Blackmon L (2010). Perinatal regionalization for very low-birth-weight and very preterm infants: a meta-analysis. JAMA.

[5] Edwards JD, Houtrow AJ, Vasilevskis EE, Rehm RS, Markovitz BP, Graham RJ (2012). Chronic conditions among children admitted to U.S. pediatric intensive care units: their prevalence and impact on risk for mortality and prolonged length of stay. Crit Care Med.

[6] McShane P, Draper ES, McKinney PA, McFadzean J, Parslow RC, and Paediatric Intensive Care Audit N (2013). Effects of out-of-hours and winter admissions and number of patients per unit on mortality in pediatric intensive care. J Pediatr.

[7] Tasker RC, Fleming TJ, Young AE, Morris KP, Parslow RC (2011). Severe head injury in children: intensive care unit activity and mortality in England and Wales. Br J Neurosurg.

[8] Typpo KV, Petersen NJ, Petersen LA, Mariscalco MM (2010). Children with chronic illness return to their baseline functional status after organ dysfunction on the first day of admission in the pediatric intensive care unit. J Pediatr.

[9] Fraser LK, Miller M, Draper ES, McKinney PA, Parslow RC, and Paediatric Intensive Care Audit N (2011). Place of death and palliative care following discharge from paediatric intensive care units. Arch Dis Child.

[10] Maybloom B, Chapple J, Davidson LL (2002). Admissions for critically ill children: where and why?. Intensive Crit Care Nurs.

[11] Australian Bureau of Statistics. Regional Population Growth, Australia, 2014–15*.* 2016;Catalogue No. 3218.0 http://www.abs.gov.au/ausstats/abs@.nsf/mf/3218.0. Accessed Dec 2016.

[12] NSW Department of Health. Paediatric healthcare. March 2017]; Available from: http://www.health.nsw.gov.au/kidsfamilies/paediatric/Pages/paediatric-healthcare.aspx. Accessed Mar 2017.

[13] NSW Department of Health. NETS - Health Care in Our Region. March 2017]; Available from: http://www.nets.org.au/About-NETS/Health-Care-in-Our-Region.aspx. Accessed Mar 2017.

[14] NSW Department of Health. The Newborn & Pediatric Emergency Transport Service (NETS). March 2017]; Available from: http://www.nets.org.au/. Accessed Mar 2017.

[15] Centre for Health Record Linkage CHeReL Quality Assurance Procedures for Record Linkage. http://www.cherel.org.au/quality-assurance. Accessed Feb 2017.

[16] Hardelid P, Dattani N, Gilbert R, Programme Board of the Royal College of P, Child H, and Child Death Overview Working G (2014). Estimating the prevalence of chronic conditions in children who die in England, Scotland and Wales: a data linkage cohort study. BMJ Open.

[17] Bentley JP, Ford JB, Taylor LK, Irvine KA, Roberts CL (2012). Investigating linkage rates among probabilistically linked birth and hospitalization records. BMC Med Res Methodol.

[18] Henderson T, Shepheard J, Sundararajan V (2006). Quality of diagnosis and procedure coding in ICD-10 administrative data. Med Care.

[19] NSW Department of Health. NSW Mothers and Babies reports 2001–2005. http://www.health.nsw.gov.au/pubs/index.asp (accessed Dec 2007).

[20] Roberts CL, Bell JC, Ford JB, Morris JM (2009). Monitoring the quality of maternity care - how well are labour and delivery events reported in population health data?. Paed Perinatal Epidemiol.

[21] Taylor L, Travis S, Pym M, Olive E, Henderson-Smart D (2005). How useful are hospital morbidity data for monitoring conditions occurring in the perinatal period?. Aust N Z J Obstet Gynaecol.

[22] Bennett TD, Spaeder MC, Matos RI, Watson RS, Typpo KV, Khemani RG (2014). Existing data analysis in pediatric critical care research. Front Pediatr.

[23] Straney L, Clements A, Alexander J, Slater A, Group APS (2010). Quantifying variation of paediatric length of stay among intensive care units in Australia and New Zealand. Qual Saf Health Care.

[24] Feudtner C, Feinstein JA, Zhong W, Hall M, Dai D (2014). Pediatric complex chronic conditions classification system version 2: updated for ICD-10 and complex medical technology dependence and transplantation. BMC Pediatr.

[25] Burns MR, Bodansky HJ, Parslow RC (2010). Paediatric intensive care admissions for acute diabetes complications. Diabet Med.

[26] Ramnarayan P, Craig F, Petros A, Pierce C (2007). Characteristics of deaths occurring in hospitalised children: changing trends. J Med Ethics.

[27] Fivez T, Kerklaan D, Mesotten D, Verbruggen S, Wouters PJ, Vanhorebeek I (2016). Early versus late parenteral nutrition in critically ill children. N Engl J Med.

[28] Armano R, Gauvin F, Ducruet T, Lacroix J (2005). Determinants of red blood cell transfusions in a pediatric critical care unit: a prospective, descriptive epidemiological study. Crit Care Med.

[29] Lacroix J, Hebert PC, Hutchison JS, Hume HA, Tucci M, Ducruet T (2007). Transfusion strategies for patients in pediatric intensive care units. N Engl J Med.

[30] Hagger-Johnson G, Harron K, Gonzalez-Izquierdo A, Cortina-Borja M, Dattani N, Muller-Pebody B (2015). Identifying possible false matches in anonymized hospital administrative data without patient identifiers. Health Serv Res.

[31] Harron K, Parslow R, Mok Q, Tibby SM, Wade A, Muller-Pebody B (2015). Monitoring quality of care through linkage of administrative data: national trends in bloodstream infection in U.K. PICUs 2003-2012. Crit Care Med.

[32] Bentley JP, Roberts CL, Bowen JR (2016). Planned birth before 39 weeks and child development: a population-based study. Pediatrics.

[33] Hennessy D, Torvaldsen S, and Roberts CL. Linkage rate between NSW Perinatal Data Collection birth records and government school NAPLAN educational records, by gestational age at birth. 2016;http://hdl.handle.net/2123/15755. Accessed Jan 2017.

